# The cost of a knowledge silo: a systematic re-review of water, sanitation and hygiene interventions

**DOI:** 10.1093/heapol/czu039

**Published:** 2014-05-29

**Authors:** Michael Loevinsohn, Lyla Mehta, Katie Cuming, Alan Nicol, Oliver Cumming, Jeroen H J Ensink

**Affiliations:** ^1^Knowledge, Technology and Society Team, Institute of Development Studies, Library Road, Brighton BN1 9RE, UK, ^2^Noragric, Norwegian University of Life Sciences, P.O. Box 5003, NO-1432 Aas, Norway, ^3^Environmental Health Group, Department of Disease Control, Faculty of Infectious Tropical Disease, London School of Hygiene and Tropical Medicine, Keppel Street, London WC1E 7HT and ^4^Present address: CARE International in Uganda, Box 7280 Kampala, Uganda

**Keywords:** Diarrhoea, multiple benefits, realist review, water supply, sanitation, hygiene

## Abstract

Divisions between communities, disciplinary and practice, impede understanding of how complex interventions in health and other sectors actually work and slow the development and spread of more effective ones. We test this hypothesis by re-reviewing a Cochrane-standard systematic review (SR) of water, sanitation and hygiene (WASH) interventions’ impact on child diarrhoea morbidity: can greater understanding of impacts and how they are achieved be gained when the same papers are reviewed jointly from health and development perspectives? Using realist review methods, researchers examined the 27 papers for evidence of other impact pathways operating than assumed in the papers and SR. Evidence relating to four questions was judged on a scale of likelihood. At the ‘more than possible’ or ‘likely’ level, 22% of interventions were judged to involve substantially more actions than the SR’s label indicated; 37% resulted in substantial additional impacts, beyond reduced diarrhoea morbidity; and unforeseen actions by individuals, households or communities substantially contributed to the impacts in 48% of studies. In 44%, it was judged that these additional impacts and actions would have substantially affected the intervention’s effect on diarrhoea morbidity. The prevalence of these impacts and actions might well be found greater in studies not so narrowly selected. We identify six impact pathways suggested by these studies that were not considered by the SR: these are tentative, given the limitations of the literature we reviewed, but may help stimulate wider review and primary evaluation efforts. This re-review offers a fuller understanding of the impacts of these interventions and how they are produced, pointing to several ways in which investments might enhance health and wellbeing. It suggests that some conclusions of the SR and earlier reviews should be reconsidered. Moreover, it contributes important experience to the continuing debate on appropriate methods to evaluate and synthesize evidence on complex interventions.

**KEY MESSAGES**

- Understanding how complex interventions achieve their effects—how they are made to work or not—is impeded by disconnects between health and other disciplinary and practice communities.
- When evaluations of water, sanitation and hygiene interventions were reviewed jointly from health and development perspectives, evidence of a broader range of impacts emerged than when they had been reviewed from a health perspective alone.
- Other actions than those assumed by the original systematic review often contributed to impacts, in some cases calling into question the conclusions of the original systematic review.
- Additional impact pathways are suggested, pointing to a number of ways in which investments in these interventions might enhance health and wellbeing.

## Introduction

It is not enough merely to know that an intervention aimed at improving health works in order to adapt it to new situations and widen its impact. Understanding how it achieves its effects—how it is made to work or not—is crucial. Particularly when the intervention involves the actions of many individuals deployed in organizations, a broader range of disciplines than the health sciences is required to generate the evidence required. Social science perspectives in particular are needed to make sense of the continuing obstacles to implementing health system interventions such as the World Health Organization essential medicines programme (Peters and Bennett 2012) or to clarify how quality improvement initiatives, such as the Michigan Keystone Project that reduced central venous catheter bloodstream infections in intensive care units, actually achieve their results (Dixon-Woods *et al.* 2011). However, these disciplines are not routinely drawn into the evaluation of complex health interventions. There is as yet no consensus on appropriate methods for such evaluations and for the synthesis of findings across evaluations (Shepperd *et al.* 2009; Petticrew *et al.* 2013; Wong 2013).

This article examines in depth the consequences of knowledge silos in three classes of interventions: water supply, sanitation and hygiene. Although all have the potential to prevent significant morbidity and mortality, the first two in particular are often not designed or implemented with health outcomes as their primary objective and are typically not the responsibility of health ministries. Yet the three are commonly referred to collectively as a sector, water, sanitation and hygiene (WASH), because of their critical contribution to health (Bartram and Cairncross 2010).

Progress in achieving broad access to WASH has been slow, particularly for sanitation. In 2011 some 2.5 billion people were living without access to improved sanitation facilities, and 770 million people were not receiving their drinking water from improved water sources, according to the Global Annual Assessment of Sanitation and Drinking-Water (The ‘GLAAS Report’). The report linked these persistent gaps to the toll of diarrhoea, the second leading contributor to the global burden of disease, and then asked a series of questions: ‘Where are the real bottlenecks? Are they in the formulation and implementation of policies? In the process of optimizing institutions and the arrangements between them? In the translation of political will into action? In the decision making on the allocation of resources at national and international levels? Or in the current education and training programmes for professionals working in water and sanitation (World Health Organization 2010)?’

We hypothesize that understanding of this problem has been undermined by the continuing disconnect between two disciplinary and practice communities: the world of health and disease transmission (in which the GLAAS report is firmly situated), and the wider development world, which generally assumes that a diversity of perspectives, interests, power and rights drives the actions that shape such complex situations (Roe 1998: Leach *et al.* 2010). The disconnect between these communities hampers comprehensive diagnosis of the problem and the mounting of more effective actions to address it.

The relationship between ill-health and poor water supply, sanitation and hygiene has been a concern of public health since the beginning of the discipline in the 19th century. Although a range of infections, parasitic, bacterial and viral, may be prevalent in such conditions (Bartram and Cairncross 2010) as well as sub-clinical disorders such as environmental enteropathy (Humphrey 2009), it is diarrhoea that has attracted most attention from public health. Diarrhoea in children under 5 years of age is responsible for ∼15% of deaths (Black *et al.* 2010) and exacerbates other leading causes of mortality and morbidity such as HIV/AIDS and measles. It is a significant risk factor in the development of malnutrition, with each additional episode in the first 24 months of life increasing the risk of stunting by roughly 5% (Black *et al.* 2008). Repeated bouts of diarrhoea also have longer term effects on the child’s physical and mental development (Guerrant *et al.* 2002).

This dominant concern with the diarrhoeal impacts of WASH interventions has been reinforced by a series of systematic reviews (SRs), the first in 1983, and the most recent in 2010 (Blum and Feachem 1983; Esrey *et al.* 1991; Curtis and Cairncross 2003; Fewtrell *et al.* 2005; Arnold and Colford 2007; Clasen *et al.* 2007; Aiello *et al.* 2008; Ejemot-Nwadiaro *et al.* 2008; Schmidt and Cairncross 2009; Waddington and Snilstveit 2009; Cairncross *et al.* 2010; Clasen *et al.* 2010; Norman *et al.* 2010). Responding to the broader calls in medicine and public health for evidence-based policy, these reviews drew on primary studies for the most part in low- and middle-income countries with the principal objective of informing the investment decisions of international assistance and national programmes in the WASH sector.

In 10 of these 13 reviews, the concern was with diarrhoea morbidity alone; in two cases, parasitic diseases (Esrey *et al.* 1991; Norman *et al.* 2010) and in one case respiratory diseases (Aiello *et al.* 2008) were also considered. Although some of the reviews discussed aspects of the institutional and technical context that influence the effectiveness of interventions—Blum and Feachem (1983), the first in the series stands out in this respect—the focus has been on the outcome of WASH interventions. From Esrey *et al.* (1991) on, this has been reflected in the reporting of effect sizes for different categories of WASH interventions, calculated by meta-analysis. Across the series of reviews, there is a trend towards more rigorous inclusion and exclusion criteria following Cochrane and Campbell Collaborations standards, with experimental and quasi-experimental evaluations of interventions increasingly judged of highest quality. Clasen *et al.* (2010), the most recent review, admitted only randomized controlled trials.

This preponderant concern with health outcomes, diarrhoea in particular, has not been balanced with SRs of the non-health benefits of WASH interventions—we have not been able to identify any—though these may be substantial. A dependable and safe water supply in or near the home allows more time for pursuits such as education, income generation, child care and leisure—women and girls often benefiting the most (Blum *et al.* 1990). Improved water supply is commonly used by householders to irrigate kitchen gardens or for other productive enterprises, enhancing income and food security (Nicol 2000; van Koppen *et al.* 2009). Poor households gaining access to a reliable public supply avoid the often exorbitant price they face for private provisioning and can divert the savings to food and other essentials (Galiani *et al.* 2008). Accessible, closed toilets or latrines are valued for the dignity they preserve, the security they provide and the time they save compared to open defecation (Kar and Chambers 2008). Girls are more likely to attend school if improved latrines are available there (Birdthistle *et al.* 2011).

These non-health benefits are significant in their own right, enabling important capabilities relevant to at least three millennium development goals. They may also enable the realization of health benefits. For example, when women are better educated, they are able to make more effective use of water and sanitation infrastructure and provide better care for their children, adapting hygiene practices they have learned about to their own situations (Jalan and Ravallion 2003). Better nourished children are able to mount more effective immune responses to infections and their development is less affected by them (Katona and Katona-Apte 2008). The need to reflect these pervasive interactions in the setting of development goals beyond the Millennium Development Goals (MDG) target date of 2015 is widely recognized (Waage *et al.* 2010; UN System Task Team 2012)

To the extent then that SRs aim at influencing policy direction and investment, there is reason to believe that the available reviews, taken together, provide an incomplete picture of the benefits of WASH interventions. Focused on the impact of these interventions, they provide limited insight into the contextual factors that can affect the achievement of impact. From a development perspective, possibly, the most striking absence is the general lack of concern with the agency of beneficiaries, individually and collectively, pursuing objectives congruent or not with those of the intervention, and of the implementing organization’s personnel who shape and adapt the intervention to local conditions. Also largely absent is analysis of the influence of interventions in other fields that affect the same areas and people, mounted by organizations that may or may not co-ordinate their actions with those that are implementing the WASH intervention. More effective harnessing of local innovative capabilities, it has been argued, is vital if the ambitious successors to the MDGs needed for the planet to remain within critical environmental and social boundaries are to be achieved (Leach *et al.* 2012).

Our objective in this article is to test the hypothesis laid out above: can greater practical understanding be gained of the overall impacts of WASH interventions and how they are achieved when examined jointly from health and development perspectives? We do this by re-reviewing the studies that were included in the most comprehensive of the recent SRs of the impact of WASH interventions on diarrhoea morbidity, the ‘Waddington review’ (Waddington and Snilstveit 2009; Waddington *et al.* 2009). The approach makes possible a direct comparison, on the same body of studies, with the findings obtained using a purely health perspective and a primarily quantitative synthesis of primary studies.^1^ At the same time, it limits the conclusions that can be drawn about the literature as a whole since that sample of evidence is biased. The papers in the Waddington review were selected for the contribution they could make to the meta-analysis of diarrhoea impact: many provide only limited description of the implementation context and of the influence of different actors.

We are aware of only one other use of this approach: a re-review of papers from a Cochrane review of school feeding programmes’ impacts on child nutrition and cognitive development (Greenhalgh *et al.* 2007).

## Methods

### Review framework

In common with the authors of that study, we employ the methods of realist review (Pawson *et al.* 2005). This holds that the outcomes produced by the underlying mechanisms an intervention unleashes depend on the context in which this occurs. We consider that most WASH interventions are inherently complex, recognizing two relevant senses of the term. The UK Medical Research Council (2000) views interventions as complex that ‘comprise a number of separate elements essential … to the proper functioning of the intervention’. More broadly, realist review recognizes several defining features of complex interventions, among which that their effects result from the actions of many individuals—implementers, intended and unintended beneficiaries, and others who influence the intervention and the context; that these actors adapt interventions to their social, political and natural environments; and that their actions can produce consequences unforeseen by the intervention’s designers, both positive and negative (Pawson *et al.* 2005).

Realist review understands interventions to be based on ‘programme theories’ that link their delivery to the desired outcome. For example, a hygiene intervention may assume, explicitly or implicitly, that diarrhoea-causing pathogens are transmitted primarily via the faecal-oral route, that handwashing with soap after defecation, after cleaning a child who has defecated and before preparing or consuming food can interrupt most of that transmission; and that by distributing soap and communicating the importance of handwashing through a series of measures handwashing will be more widely practised, which will significantly reduce diarrhoea morbidity. Realist review casts each element in this programme theory in terms of the contextual influences (C) (e.g. mothers engaged in a locally developed educational process) that trigger a mechanism (M) (learning hygiene skills and understanding their importance) that results in the desired outcome (O) (mothers washing their hands after defecation or cleaning a child and before preparing food). These are referred to as C-M-O configurations.

The articles included in the Waddington review generated evidence to test their programme theories, at least the overall relationship between intervention and outcome and, in some cases, one or more of the intermediate elements as well. The methods employed by the Waddington review, the results of their literature search and details of the papers included can be found in Waddington and Snilstveit (2009).

In our re-review, we searched for evidence of other impact pathways operating in addition to those assumed in the papers’ programme theories and the Waddington review. We evaluated evidence that had been noted by the study authors, in textual or numeric form, and in some cases commented on.

Our review developed in an iterative fashion. Our initial focus was on evidence of multiple benefits or harms, beyond reduction of diarrhoea morbidity. However, as we engaged with this literature, we became aware that other impact pathways were evident as well and that in some cases they operated not in parallel to the diarrhoea reduction pathway assumed by the authors but rather interacted with it. We eventually settled on three questions relating to pathways and one overarching question that enabled us to test our hypothesis in some depth:

1. Is the intervention substantially more complex than considered by the Waddington review? Taking ‘complex’ in the narrow sense, relevant here are actions by the responsible organization or other actors that might have affected the impacts experienced and that went beyond what would normally be part of a WASH intervention.
2. Are the intervention’s impacts substantially understated if only the diarrhoea morbidity outcome is considered? Relevant here is evidence of benefits or harms from the intervention other than reduced diarrhoea.
3. Are actions by individuals, households or communities substantially influencing the impacts experienced? Relevant here are actions that go beyond what is assumed in the intervention’s programme theory e.g. householders doing more than just using a latrine provided through a sanitation intervention.
4. Would these other impacts and actions substantially affect the level, distribution or sustainability of the diarrhoea morbidity outcome? Relevant here is evidence of effects on the diarrhoea outcome itself or on its estimation.

### Review methods

Our methods are in line with the recently developed Realist And Meta-narrative Evidence Syntheses: Evolving Standards (RAMESES) publication and quality standards for realist reviews (Wong *et al.* 2013; Wong *et al.* n.d .). As discussed above, our initial search of the literature made use of the one done by the Waddington review. This enabled us to test the hypothesis that greater practical understanding can be gained of the overall impacts of WASH interventions and how they are achieved when studies are reviewed jointly from health and development perspectives. The test would not have been as clear-cut had different sets of studies been reviewed. The RAMESES standards countenance this departure from common practice: ‘Searching should be guided by the objectives and focus of the synthesis …’ (Wong *et al.* 2013).

We applied two exclusion criteria to the 65 studies that Waddington *et al.* reviewed. We excluded studies that provided extremely limited descriptions of the implementation context and studies that gave people very restricted space in which to exercise agency. On the first criterion, we excluded six reports of national-scale programmes. These gave very few details of either the setting of the intervention or of the intervention as it was actually implemented. On the second criterion, we excluded 27 studies of point-of-use (POU) water quality interventions. These were generally experimental, researcher-led studies (as opposed to evaluations of operational programmes) in which people were offered material (filters, flocculants or chlorine) free that was not available in local markets. Their only choice was essentially either to use it or not. Five studies were excluded for other reasons: three were only available as abstracts and two were unavailable in English.

The remaining 27 studies describe research conducted between 1982 and 2009. Six were classified by the Waddington review as randomized or cluster-randomized controlled trials and 19 as non-randomized controlled trials. Seven were assessed as of low quality by the Waddington review, most commonly because control and treatment groups were not sufficiently comparable or because the length of recall of diarrhoea episodes by caregivers exceeded 2 weeks. Thirteen described hygiene, four water supply, three sanitation and seven multiple interventions. These studies were included in our re-review and are listed in supplementary Appendix S1.

We employed an essentially forensic approach in our re-review. The studies were divided among three teams of two, drawn from the authors, one of whom had a predominantly health research background, the other a predominantly development research background. The two independently reviewed the studies using a pre-designed form (supplementary Appendix S2). This asked them to identify and extract evidence relevant to the four questions. They then judged the quality of the evidence on a three-point scale. How likely was it that the correct answer to the question was affirmative?

- Possible (substantial additional evidence needed)
- More than possible (some additional evidence needed)
- Likely (little or no additional evidence needed).

A consensus on these judgments was reached through deliberation between the two reviewers. The lead author, who read each paper, ensured that similar evidentiary standards were used by discussing with the three groups the evidence uncovered and the judgments based on it.

A further stage of deliberation followed when the studies had all been reviewed. We looked across the studies for regularities of outcome and for the ‘middle range’ and broader theories that could explain them. Middle range theories are ‘specific enough to generate propositions that can be tested about aspects of the program but sufficiently abstract to be applicable to other programs’ (Wong *et al.* 2013). We then described these regularities and explanations in terms of C-M-O configurations; these represent impact pathways.

**Figure 1:**
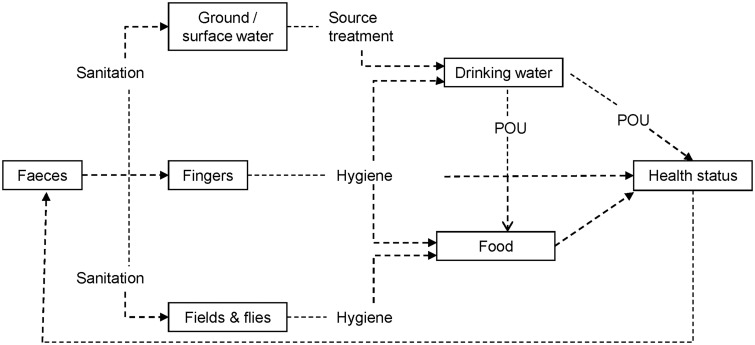
Water treatment, sanitation and hygiene barriers to disease transmission (Waddington and Snilstveit 2009). Reprinted by permission of the publisher (Taylor & Francis Ltd, http://www.tandf.co.uk/journals).

The broader theory we drew on to help explain these regularities included the following:

- Water supply, sanitation and hygiene interventions act at different points along faecal-oral transmission paths (Figure 1; Waddington and Snilstveit 2009) and may interact synergistically to reduce exposure to pathogens (Bartram and Cairncross 2010; Mara *et al.* 2010).
- Access to food, adequate in quantity and quality, is a necessary but not sufficient condition for good nutrition. Reducing malnutrition and specific micronutrient deficiencies improves immune function, strengthening resistance to infections, including diarrhoea (UNICEF1990; Katona and Katona-Apte 2008).
- Women’s status, education and access to information are powerful influences on children’s wellbeing, including their nutritional status and likelihood of receiving good health care (Smith *et al.* 2003). Education enables women to make better use of water and sanitation infrastructure, thereby gaining health benefits for their children (Jalan and Ravallion 2003).
- To sustain a livelihood, people draw on the natural assets that they have access to, notably water, combining these with their human endowments and capabilities and the financial and social capital which they can claim (Chambers and Conway 1992; Scoones 1998; Nicol 2000).
- The emergence of effective local governance of natural resources, such as water, without the need for government regulation, is favoured by particular features of the social, economic and ecological context (Ostrom 2009). This common property framework can also be used to assess the prospects of hazards, including those created by human waste, being effectively managed at the local level.

## Results and discussion

At the ‘more than possible’ or ‘likely’ level, in 37% of the studies substantially more impacts, positive or negative, were judged to have resulted from the intervention than only reduced diarrhoea morbidity (Table 1). In 48% of the studies, actions by individuals, households or communities that were not foreseen in the intervention’s programme theory were judged to have substantially contributed to the impacts experienced. In 44%, it was judged that these additional impacts and actions would have substantially affected the level, distribution or sustainability of the intervention’s effect on diarrhoea morbidity, contributing materially to that impact or influencing its estimation.

**Table 1:** Reassessment of studies from the Waddington review

	Is the intervention substantially more complex than considered by the Waddington review?	Are impacts substantially understated if only diarrhoea outcome is considered?	Are actions by individuals, households or communities substantially influencing the benefits and harms experienced?	Would these other impacts and actions substantially affect the level, distribution or sustainability of the diarrhoea outcome?
**Likely**	5 (18.5)	6 (22.2)	5 (18.5)	5 (18.5)
**More than possible**	1 (3.7)	4 (14.8)	8 (29.6)	7 (25.9)
**Possible**	4 (14.8)	1 (3.7)	3 (11.1)	4 (14.8)
**No evidence**	17 (63.0)	16 (59.3)	11 (40.7)	11 (40.7)

*Note: N *= 27. Data are number (%).

A smaller proportion of studies, 22%, were judged to be substantially more complex than described by the Waddington review. However, the difference with the other review questions was not significant (*P *= 0.21). The evidence that we found for other impact pathways operating had been integrated in the conclusions of 4 of the 27 studies (Tonglet *et al.* 1992; Haggerty *et al.* 1994; Bateman *et al.* 1995; Kremer *et al.* 2009) but none was apparently drawn on by the Waddington review.

The four review questions were not significantly more likely to be judged affirmatively at the more than possible or likely level in studies published before the median (1996) than in those published after or in studies considered by the Waddington review to be of high or low quality. They were less likely to be judged affirmatively in studies where hygiene was the only element of the intervention compared with ones where water, sanitation or multiple elements were involved. However, the difference was of borderline significance (*P *= 0.07, with Bonferroni’s correction *P *= 0.24) only for the second question where the frequency of affirmative judgments was almost four times greater in non-hygiene intervention studies (Table 2). The relatively small number of papers re-reviewed makes it difficult to distinguish such heterogeneity.

**Table 2:** Reassessment of hygiene interventions in the Waddington review: prevalence of affirmative judgments at the ‘more than possible’ or ‘likely’ level

	Is the intervention substantially more complex than considered by the Waddington review?	Are impacts substantially understated if only diarrhoea outcome is considered?	Are actions by individuals, households or communities substantially influencing the benefits and harms experienced?	Would these other impacts and actions substantially affect the level, distribution or sustainability of the diarrhoea outcome?
**Hygiene only (*n* = 13)**	2 (15.4)	2 (15.4)	4 (30.8)	4 (30.8)
**Other interventions (*n* = 14)**	4 (28.6)	8 (57.1)	9 (64.3)	8 (57.1)

*Note:* Data are number (%).

Hygiene studies, many focused on handwashing, are often similar to point-of-use water quality interventions in providing limited scope for people’s agency. As noted earlier, the sample of studies we re-reviewed generally pays little attention to agency, the implementation context and impacts other than diarrhoea morbidity reduction. The absence of evidence in these respects that we find in the majority of studies cannot be taken as evidence that these features are absent in the situations they describe. The results suggest that research perspectives and designs more open to these influences and effects would likely find evidence of them in greater proportions than we report.

In the following sections we examine the evidence supporting affirmative judgments and the impact pathways, summarized as realist C-M-O configurations, that this evidence suggests. These pathways must be considered tentative given the narrow base of studies, pre-selected for a different kind of review, on which they are based. However, they may be of use in stimulating future review and primary evaluation efforts in this area which can refine them.

To avoid confusion and double counting, we describe evidence in individual studies in relation to the particular question that it directly relates to even though it may be relevant as a consequence to another question. For example, people’s actions may be substantially altering an intervention and thereby creating additional benefits beyond diarrhoea reduction. However, we will discuss it together with other studies bearing on the first question.

### Are interventions more complex?

In two studies we found clear evidence of the interveners doing substantially more than suggested by the Waddington review’s classification. Torun (1983) describe an educational campaign in a Guatemalan village that comprised 11 topics including nutrition (promotion of weaning foods, breastfeeding and agricultural diversification), the recognition and treatment of diarrhoea as well as hygiene issues. However, only hygiene behaviours were monitored and discussed in relation to diarrhoea reduction. Although the nutrition and care elements might also have influenced behaviours and diarrhoea, there was no evidence to support this. However, we judged that the intervention was likely more complex than ‘hygiene education’, as the Waddington review described it.

Moraes *et al.* (2003) describe what the Waddington review refers to as a sanitation intervention, comprising sewerage and drainage, in Salvador, Brazil. The study authors conclude: ‘… improved environmental sanitation can have a positive impact on diarrhoeal morbidity in young children in poor peri-urban areas such as those studied’. However, the intervention Moraes *et al.* describe involved, in addition to extending drains and sewer connections, paving streets, granting residents title to their home plots and improving the water distribution system. More households in the intervention areas enjoyed a regular water supply (70% where both drains and sewerage were installed vs 38% in the control neighbourhoods) and greater consumption (71 l/capita/day vs 39 l/capita/day). We judged it likely both that the intervention was more complex than just sanitation and that substantial additional benefits were realized.

The study authors considered improved water supply to have been an independent impact of the intervention and so did not correct for these differences between neighbourhoods in their analysis of the intervention’s impact on diarrhoea. However, there is a good reason to believe that the improved reliability of access perhaps as much as the increased quantity of water consumed would have contributed to the reduction in diarrhoea (Howard and Bartram 2003; Hunter *et al.* 2010). The improvement in the water supply appears to have been long lived, since the assessment was carried out several years after the intervention. We therefore judged it more than possible that the study and the Waddington review overstated sanitation’s impact on diarrhoea morbidity.

In four studies, there is evidence of unco-ordinated actions by different agencies in sequence, which may render delineation of ‘interventions’ problematic and attribution of impact hazardous. Here we describe two of these studies. Luby *et al.* (2004) present a hygiene intervention in Karachi in which soap was distributed to households and its use promoted. Their baseline data show that in the neighbourhood where the treatment was to be implemented, the prevalence of soap purchase was substantially higher (64% of households) than in the other two (53% and 9%). These large initial differences suggest a prior effort to affect the situation, creating a more complex intervention than assumed by the Waddington review. Moreover, the high levels of soap purchase suggest people’s independent actions were contributing to the health benefits experienced and thereby reducing the impact on diarrhoea that could be attributed to the current intervention. This affects both the study’s and the Waddington review’s conclusions. It is not clear whether the statistical procedure employed, generalized estimating equations (GEE), would have been able to control for the baseline difference in soap use, but it would not have taken account of the differences in attitude towards soap use that the data suggest.

In Tehran, neighbourhoods connected to the urban sewerage system in the first phase of an expansion programme were compared with those scheduled for connection in a later phase (Kolahi *et al.* 2009). The authors collected information on socio-economic variables and diarrhoea incidence prior to the first connections and again 5 years later. Incidence declined 46% in the treatment and 37% in the control neighbourhoods, a difference which the authors attribute to the sanitation intervention.

However, reanalysis of the socio-economic data indicates that, over the period, mothers’ educational attainment in the treatment neighbourhoods improved significantly (*P* < 0.001), with 25% fewer women having a primary education or less and 40% more achieving a diploma or higher. No such improvement was seen in the control neighbourhoods. These data suggest an independent educational effort that affected some areas but not others, creating a more complex intervention than indicated by the Waddington review. Women in the treatment neighbourhoods would thereby have achieved an important additional benefit. Moreover, better educated mothers would be expected to make better use of the improved sanitation, as discussed above, contributing to reduced diarrhoea in their children. This would undermine the attribution of the decline in diarrhoea morbidity to the intervention alone claimed by the authors and assumed by the Waddington review, but it also suggests a programmatic synergy with policy implications. Jalan and Ravallion (2003) draw from their study on rural water supply in India ‘the importance of combining public investments in this type of infrastructure with other interventions in education and income-poverty reduction’. The limited statistical analysis, the absence of social science input and the apparent failure to follow up on the Tehran study means that an important opportunity to advance understanding has been lost.

Table 3 summarizes two related impact pathways these cases suggest. The studies in pathway (a) lend support to Pawson *et al.*’s (2005) caution to systematic reviewers to beware of ‘label naiveté’: the title an intervention carries speaks to ‘a general and abstract programme theory that differs from the one practitioners and managers have implemented and empirical studies have evaluated. Broadly speaking, then, we should expect the same intervention to be delivered in a mutating fashion, shaped by refinement, reinvention and adaptation to local circumstances’.

**Table 3:** Impact pathways related to intervention complexity

Context	Mechanism	Outcome	Implication for the diarrhoea outcome or its estimation in the study and the Waddington review
Agencies make operational decisions on what to include in their intervention and where to intervene.	(a) Staff modify intervention in response to local circumstances (Torun 1983, Moraes *et al.* 2003)	(a) The intervention implemented differs substantially from the label, involving additional elements that affect its impact	Effect of the (current) intervention is overestimated.
	(b) Interventions cluster in certain areas to draw on the information from earlier efforts or in response to policy or administrative directive (Bateman *et al.* 1995; Luby *et al.* 2004; Garrett *et al.* 2008, Kolahi *et al.* 2009).	(b) What people experience includes the contribution of both the current and earlier interventions.	

The succession and clustering of interventions that we see in at least four cases (pathway b) does not appear to be an isolated occurrence. ‘Programs are the offspring of previous interventions. Social problems are longstanding; interventions evolve to try to combat them; the success of a current scheme depends on its history’ (Pawson *et al.* 2011). ‘[M]ultiple overlapping interventions occur in poorer areas simply because this is how policies are often intentionally targeted’ (Petticrew *et al.* 2005).

### Are more impacts realized?

We found evidence in several studies, suggesting that the intervention had made possible substantial benefits in addition to diarrhoea reduction. In Buenos Aires, a water supply intervention enabled shantytown households to connect to the urban system, increasing the quality and reliability of the water they could access, contributing thereby to reduced diarrhoea burdens (Galiani *et al.* 2008). They also saved time that had been spent in fetching water and money spent on procuring water.

In rural Nigeria, Huttly *et al.* (1990) noted time savings, reduced morbidity due to dracunculiasis (guinea worm), and declining prevalence of wasting following a water supply and sanitation intervention. Reduced dracunculiasis incidence was also noted by Gasana *et al.* (2002) in rural Rwanda after improvements to the water supply. These benefits are to be expected: as noted earlier, WASH interventions interrupt transmission of a number of infections, alleviate malnutrition and save people time lost due to illness and in seeking water or a place to defecate (Bartram and Cairncross 2010). Privacy and dignity may also be enhanced by improved and accessible sanitation.

Table 4 summarizes this impact pathway. Such multiple benefits might be expected to increase people’s commitment to support or maintain these interventions; however, there is no evidence bearing on this in these studies.

**Table 4:** Impact pathway related to the direct multiple benefits of interventions

Context	Mechanism	Outcome	Implication for the diarrhoea outcome or its estimation in the study and the Waddington review
Several sources of ill-being, including non-diahorreal infections, are linked to poor access to water and to insanitary environment.	WASH interventions alleviate determinants of these different sources of ill-being.	Multiple benefits (health, time and expense saved by more accessible services), in addition to diarrhoea reduction, may be realized as a direct consequence of the intervention (Huttly *et al.* 1990; Gasana *et al.* 2002; Galiani *et al.* 2008).	Possibly no effect (additional benefits are valued in their own right); may increase beneficiaries’ commitment to supporting and maintaining the intervention, enhancing sustainability.

There is evidence as well of interventions unintentionally doing harm. In two studies, existing inequality in access to services and possibly health status appears to have been exacerbated by the intervention’s siting. In the Salvador study mentioned earlier (Moraes *et al.* 2003), control neighbourhoods were those left uncovered by the sanitation programme when funds ran out. Priority was to have been given to neighbourhoods that lacked access to basic services, had low average income or were vulnerable to flooding or landslide, among other characteristics. However, in practice, the authors observe, political patronage and pressure from construction firms preferring to work in the easiest terrain influenced the allocation. No baseline socio-economic or health data are available, but the survey 5 years after implementation shows that household income and levels of schooling were significantly lower and the proportion of recent rural immigrants higher in the control areas. Neighbourhoods of higher socio-economic status therefore apparently benefited disproportionately from the improved sanitation, water supply, paving of roads and land titling, worsening inequality. Moreover, if it is assumed that diarrhoea morbidity was greater at baseline in the lower socio-economic status control neighbourhoods, which we judge more than possible, then attributing the entire decline in diarrhoea morbidity in the treatment neighbourhoods to the intervention is unjustified.

In eastern DR Congo (DRC), a water supply intervention appears to have had a similar effect (Tonglet *et al.* 1992). Diarrhoea incidence declined significantly in villages to which piped water was delivered compared with those relying on existing sources. The pipes were sited near main roads, apparently for ease during construction, which is also where higher socio-economic status households were concentrated. Tonglet *et al.* are among the few authors who draw out the implications of the evidence for alternative impact pathways: ‘… accessibility to public standpipes is much better for the few, well-educated and best housed people, than for the many who are poorly educated and poorly housed. It is likely that the same better-off people who are the least exposed to the risk of diarrhoea, are benefiting the most from the water supply intervention’. There was no indication that this very visible, village-scale unfairness was to be mitigated in a subsequent phase.

Table 5 summarizes the related impact pathway. Lack of transparency and accountability and corruption in the provision of water and sanitation services remain serious problems in many countries. Both public and private sector providers have been implicated (Davis 2004; Hunter *et al.* 2010). Wilkinson (2006) suggests that aggravation of local inequality as in the DRC example may be particularly corrosive of wellbeing.

**Table 5:** Impact pathway related to unintended negative consequences of intervention

Context	Mechanism	Outcome	Implication for the diarrhoea outcome or its estimation in the study and the Waddington review
Agencies make operational decisions on where to site interventions and where to work first in situations of limited transparency and accountability.	These decisions may be affected by political influence, corruption and ease of access. Wealthier and healthier groups generally have greater influence and ability to offer bribes and live in more accessible, salubrious areas.	Interventions exacerbate existing inequality in services and health status.	Intervention’s effect is overestimated when comparing treatment and untreated areas without correction for baseline differences (Moraes *et al.* 2003); anti-poor distribution of benefits (Tonglet *et al.* 1992; Moraes *et al.* 2003).

Three studies describe sanitation interventions that introduced payment for latrine construction. We saw a potential for exclusion of poorer households but no specific evidence of that occurring (Huttly *et al.* 1990; Pattanayak *et al.* 2007; Garrett *et al.* 2008).

### Are individuals, households or communities influencing impacts?

In several studies, there is evidence of unanticipated benefits flowing from the actions of people affected by the intervention. Aziz *et al.* (1990a,b) and Hoque *et al.* (1996) studied a rural water supply initiative in Bangladesh and describe how women used the water not only in their homes but also to irrigate their home gardens. This began spontaneously, they write, as soon as the system was installed, in all seasons and in larger volumes than women in the control areas where water was less available. The authors say nothing further about what the women and their families gained from this unplanned use of the water supply, though, as indicated above, it is a common occurrence in rural and peri-urban contexts. They note, however, that the women valued the increased quality of life they now enjoyed more than the reduced diarrhoea burden and that this increased their commitment to maintain the system. We judged it possible that child nutrition would have improved, contributing to the decline in diarrhoea morbidity, and more than possible that the benefits gained from irrigation contributed to women’s commitment, enhancing the sustainability of the intervention.

In the Buenos Aires water supply intervention described above (Galiani *et al.* 2008), households diverted two-thirds of their monetary savings to food and beverage. We judged it possible that increased food intake would have impacted on children’s diarrhoea via improved nutrition but likely that the reduction in diarrhoea, however produced, would have had a markedly pro-poor bias, since these householders were among the most marginalized of the city’s residents.

Table 6 outlines a plausible impact pathway. Poor urban consumers tend to use a large proportion of their marginal income for food. Increasingly in many cities, they use available water supplies for small-scale agriculture, the harvest either consumed or sold primarily to purchase food (Kutiwa *et al.* 2010). Rural households employ water from domestic sources for food production in kitchen gardens, supplementing what they are able to access from irrigation and enhancing food security (van Koppen *et al.* 2009). When water supply interventions increase people’s access to water, the impact on diarrhoea via improved nutrition may be difficult to disentangle from that due to increased water consumption. The ability to use water for different purposes is likely to be highly valued by beneficiaries.

**Table 6:** Impact pathway linking domestic water supply to food production or purchase

Context	Mechanism	Outcome	Implication for the diarrhoea outcome or its estimation in the study and the Waddington review
Water supply interventions enable beneficiaries not only to avoid water-related diseases but also to access a resource, increasingly in demand, that can be used for a range of purposes.	People often use water for production (esp. of food in rural/peri-urban areas); or to reduce private expenditure for water. The poor use the additional income in large proportion to purchase food. They also save time for procuring water.	Increased food, water and time are valued in their own right. Improved child nutrition may also contribute to reduced diarrhoea.	Attribution of diarrhoea reduction solely to direct effect of water supply may be mistaken; people’s commitment to support and maintain the system is increased, enhancing sustainability (Aziz *et al.* 1990a,b; Hoque *et al.* 1996); markedly pro-poor distribution of benefits is unrecognized (Galiani *et al.* 2008).

A programme of spring protection in western Kenya improved water quality and reduced childhood diarrhoea in households, drawing their water from these springs compared with people relying on springs that were to be protected in a later phase (Kremer *et al.* 2009). Some of these comparison households began to use the protected springs, despite the greater distance they had to travel to access them, and their children benefited as well from reduced diarrhoea. Kremer *et al.* reported no evidence that the quantity or quality of the water available to the springs’ original users was affected, suggesting that the comparison households had expanded the immediate term benefits of the intervention (see further below). However, in doing so, they reduced the estimated impact of the intervention on diarrhoea.

The authors were aware of this effect and, in their Supplementary Appendix Table II, employ a different statistical procedure to estimate the reduction in diarrhoea had users drawn only on their regular spring. In at least three other studies, there is evidence of interventions spreading to the control group through the actions of people in the treatment group, In the Guatemalan educational intervention discussed earlier, all 27 of the hygiene practices monitored increased in control households, and the proportion of these households in which more than half the behaviours were deemed adequate rose 120%, compared with 560% in the treatment households (Torun 1983). The authors attribute the increase in the control households to ‘interaction and communication’ among the villagers. Shahid *et al.* (1996), in their study of a programme promoting soap use in peri-urban Dhaka, acknowledge that hygiene messages may have been spread between the adjacent treatment and control communities, although they provide no evidence of this actually happening. Bateman *et al.* (1995), in a hygiene education programme in rural Bangladesh, find more direct evidence of such spread. Both discuss the impact this would have, reducing the apparent effect of the intervention.

Controlling for ‘contamination’ between treatment and control groups is one of the quality criteria considered by the Waddington review, although contaminated impact estimates appear to be used in their meta-analysis, e.g. from Kremer *et al.*^2^ However, there is a more important point. The spread beyond the intended target group of ideas or of access to valued infrastructure and of the benefits they confer is being viewed primarily as an estimation problem, obscuring the potential it offers to enhance the reach and sustainability of interventions. Rather than considering how people’s agency might be enlisted, concern focuses on designing out its effects by greater separation or controlling for them statistically.

Table 7 outlines a relevant impact pathway. The diffusion of innovations among potential users is a subject discussed in several disciplinary literatures, much of it drawing on the seminal work of Everett Rogers (Rogers and Shoemaker 1971; Rogers 2003). Programmes have drawn on that capacity to hasten the spread of new technologies, e.g. in farmer education programmes (Van den Berg and Jiggins 2007).

**Table 7:** Impact pathway related to the diffusion of innovations

Context	Mechanism	Outcome	Implication for the diarrhoea outcome or its estimation in the study and the Waddington review
Interventions are implemented in communities whose members are linked in social networks; they are also linked, generally less intensively, with people in neighbouring communities.	Information that people gain from interventions and their experience with new practices moves through these networks.	Information from interventions or direct access to infrastructure benefits people in control as well as treatment groups.	Estimates of diarrhoea morbidity reduction based on the difference between treatment and control groups are biased downwards (Torun 1983; Bateman *et al.* 1995; Shahid *et al.* 1996; Kremer *et al.* 2009).

Haggerty *et al.* (1994) propose another explanation for a marked decline in diarrhoea morbidity in the control group of their study of a hygiene intervention in DR Congo: the intense attention to diarrhoea that accompanied the trial raised awareness and stimulated villagers to take up actions that they knew were effective against diarrhoea—a Hawthorne effect. This would again reduce the estimated impact of the intervention. The authors give no hint as to what those effective actions might be, surely important knowledge for the design of locally adapted interventions. Substantial declines in microbial contamination or diarrhoea morbidity in control groups were observed in several other studies but were generally little discussed (Lee *et al.* 1991; Pinfold and Horan 1996; Kolahi *et al.* 2009).

Most of the studies in the Waddington review focus on individuals and households and the effects of interventions in relation to their characteristics. Neighbourhoods, villages or communities typically are considered only in relation to the sampling frame, the place where individuals or households are encountered. A few studies, however, consider how characteristics of the higher level can shape the risk environment.

In Kenya, the protected springs that Kremer *et al.* (2009) studied are maintained by local committees of users, organized by the agency that undertook construction. Members are expected to pay the costs of maintenance, but in practice there is a good deal of free-riding—people using the springs without contributing to their upkeep. Indeed, that free-riding is the source of the expanded short-term benefits discussed above, but these benefits cannot be sustained without a continuing investment of cash or labour. The authors model alternative property rights regimes and conclude that the current open-access regime provides greater social benefits than any alternative based on privatizing the springs. It is not clear whether they considered the willingness of committee members to continue subsidizing maintenance in the face of rampant free-riding or the possibility of other forms of governance emerging.

Drawing on the common property framework mentioned earlier, Kremer *et al.*’s description of the situation suggests that the committee’s lack of rule-setting autonomy, constrained by tradition and policy, is a major impediment to the emergence of effective local governance. On the other hand, several features appear favourable, among them that a spring’s users are visible, users are generally known to each other—although a high-quality spring attracts users from some distance—and their actions are obvious. It would be critical to know whether the consequences of failing to maintain the springs are currently sufficiently evident to users that they would support a stricter imposition of maintenance contributions. A more detailed understanding of the situation is needed but on the available evidence, the prospects for generating sustained benefits from these protected springs appear to be feasible if the constraint on the committee’s autonomy can be overcome through focused local and political action.

Strong social norms can be an important support for local governance, and there is evidence that in some circumstances they can evolve fairly quickly. Pattanayak *et al.* (2007) describe the emergence of village-level governance through the development of norms against open defecation in a sanitation intervention in Odisha, India. Related processes are being supported on a broader scale through Community Level Total Sanitation (CLTS), an approach now being implemented in some 50 countries (CLTS Foundation, n.d.). Through experiential learning, villagers come to understand the many ways in which they and their children are contaminated by open defecation, which often triggers disgust and shame. Buttressed by social persuasion, villagers are then compelled to construct latrines or toilets, depending on their possibilities, improving the quality over time. Support from within and outside the community has often proved crucial for the poorest to improve their facilities.

The immediacy of the hazard from open defecation, the visibility of people’s actions and the fact that those responsible are known to others—more so than in the case of the Kenyan springs—are features that favour the emergence of effective local governance. Among the constraining factors are the marked social divisions in some villages (Mehta 2011) and the invisibility of some of the consequences of people’s actions, e.g. inadvertent contamination of groundwater due to poor siting of latrines leading to second-generation hazards (Dyalchand *et al.* 2011). The health impacts of CLTS have yet to be comprehensively assessed, although it is evident that people realize a range of benefits such as dignity, privacy, security—especially for women—and a clean environment, which they may value more than protection from infection (Evans *et al.* 2009; Pattanayak *et al.* 2009; Mehta and Movik 2011).

Norms and social action do not operate only in rural areas. In the Salvador study, Moraes *et al.* (2003) note that if the health impact of sanitation infrastructure is considered only at the household level, there is a risk of overlooking ‘the amplification of impact, which is likely to result when a whole community benefits from sanitation improvements, and the important degree to which diarrhoea is transmitted in the public environment’. The implication appears to be that drains and sewerage connections on your neighbours’ lots benefit your children, e.g. when they play there or in the adjacent roads and public spaces. However, the authors do not discuss the contribution of social action to this reduction of risk. The evidence for it in the paper is only suggestive. Their Table 1 indicates that house plots were markedly cleaner in treatment compared with control neighbourhoods: fewer had excreta disposed of openly and fewer had sewerage or rubbish visible within 10 m of the houses. Yet rubbish collection was not part of the intervention. Was it organized collectively? It would have been difficult to manage individually. Perhaps the most that can be said is that residents of the treatment neighbourhoods appear to have gained collectively—from a more salubrious environment with covered drains and paved roads—as well as individually, and would have been motivated to protect that space. Table 8 outlines the relevant impact pathway.

**Table 8:** Impact pathway related to local institutions

Context	Mechanism	Outcome	Implication for the diarrhoea outcome or its estimation in the study and the Waddington review
The communities in which interventions are implemented have an adaptive capacity for self-governance.	Local institutions, formal and informal, influence the spread, adaptation and retention of interventions.	Distribution of benefits and their sustainability are generally positively affected.	The effect of institutions on distribution and sustainability of the diarrhoea morbidity reduction is largely unrecognized by the SR (Pattanayak *et al.* 2007, 2009; Garrett *et al.* 2008; Kremer *et al.* 2009).

## Conclusions

We emphasize that our re-review of the Waddington review in no way implies criticism of those authors’ work: they adhered to Cochrane and Campbell Collaboration standards and went further than most previous reviewers in considering aspects of the interventions’ programme theories and implementation contexts. Our objective has been to assess what additional insights may be gained when the same body of studies is jointly reviewed from health and development perspectives, using a realist framework open to evidence of different impact pathways. We believe this openness is essential given the diversity of actors in and around WASH interventions and of their interests. The study contributes important experience to the debate in health (Shepperd *et al.* 2009; Petticrew *et al.* 2013; Wong 2013), and more widely, on appropriate methods to synthesize knowledge on complex innovations.

The re-review has provided an enlarged view of WASH interventions and their contexts. There is evidence that other interventions, previous or concurrent, sometimes influence the field in which the intervention and the evaluation operate. Multiple impacts, positive or negative, unforeseen by the intervention’s designers, may be produced, affecting health and livelihood, many of them created or shaped by beneficiaries or by people beyond the intended reach of the intervention.

The findings indicate that these effects do not always operate independently of the impact pathway assumed in an intervention’s programme theory: in many cases, they appear to be affecting, positively or negatively, the level, social distribution or sustainability of the key outcome, diarrhoea morbidity, or its estimation. It is important to note that evidence of these unaccounted for effects is common in studies that have previously been reviewed, several in more than one SR, suggesting that the apparently safe conclusions drawn from them need to be revisited.

These effects have been found in evaluations of sanitation, water supply and, possibly less frequently, hygiene interventions in a range of low- and middle-income countries. The evidence suggests a number of additional impact pathways, which in turn point to ways in which investments in these interventions can be managed to provide greater net benefits, e.g. by making provision for the productive use of water (Table 6), enlisting people’s agency in the spread of interventions (Table 7) and supporting local governance (Table 8). These suggestions are still tentative given the limited literature we reviewed: much more can likely be gleaned from studies not preselected as these have been, a point to which we return below. However, our findings support the hypothesis that valuable practical insights can be gained by bringing health and development perspectives together to investigate these interventions, shedding light on at least a part of the conundrum evoked in our introduction.

Our re-review has inherent limitations. It provides no measure of the strength of the effects it reports and relies on a qualitative assessment of their likelihood. As discussed above, the studies we draw on had been selected for their ability to contribute to a meta-analysis of WASH interventions’ impact on diarrhoea morbidity; thus, our re-review gives no insight into the prevalence of these effects in studies more open to such evidence and able to assess the evidence more closely. However, that we find evidence of these effects to be common in this sample of studies suggests that it would likely be found even more so in literature not pre-selected in this manner. We have documented our methods, which are in line with the practice of realist review, and described our reasoning, making it possible for others to follow up on our work. 

We draw implications from our study at three levels.

### More comprehensive SRs

Taking account of the limited and biased sample of studies in our re-review and of the imperative described in the introduction for more joined-up policy across sectors, we recommend that donors and commissioning organizations support one or possibly more SRs of literature on the different and multiple impacts of WASH interventions on health and livelihoods. These reviews should examine studies of different research designs, experimental and observational, in order to shed light on the various pathways through which impacts are achieved and the conditions that make possible realization of multiple benefits or that help in recognizing and responding to harms. Employing mixed quantitative and qualitative methods, they would illuminate not just average effects but also the conditions under which exceptional results are realized. These SRs would also play important roles in mapping the current literature that is fragmented among the disciplinary and practice communities, and in creating demand for evaluations that can better inform both policy and practice.

### More realistic evaluations

The papers re-reviewed here demonstrate that there is an urgent need for studies that can take the measure of operational situations as they exist. As seen above, the spread of information or of access to infrastructure to people outside the treatment group is now too often an inconvenient reality for experimental evaluations, biasing impact estimates, while its potential developmental and epidemiological significance is ignored. Greater separation of treatment and control groups can reduce contamination—though not spread itself—but provides no insight into the phenomenon and how it might be drawn on. Rather than attempting to fit situations to experimental designs, designs should be adapted to that reality: e.g. the size of the control group can be increased to allow for a ‘spread group’ whose use of the information it receives and the outcomes realized can be assessed alongside those of the treatment group and the remainder of the control group. That increased size will have cost implications.

Our re-review has highlighted the importance of bringing different perspectives to bear in assessing the evidence on what actually happens in complex innovations. Thirty years ago, Blum and Feachem (1983) urged that studies in operational settings focus on the ‘intervening processes’ between WASH interventions and their impacts, many of which involve behaviour and usage, and thus require the skills of social scientists, alongside those of engineers and epidemiologists. The need for such inter-disciplinary collaboration remains as pressing.

Beyond having the skills to follow up on the intervening processes (what we have called pathways) assumed to link intervention and impact, evaluation teams should have the flexibility to pursue evidence of other impact pathways that emerges, e.g. when substantial improvements are observed in control groups. Too often study authors were left to speculate on an unexpected result. In a number of cases, good research practice would have helped to draw more insights from the studies. A large proportion did not have baseline information on diarrhoea morbidity (let alone other impacts), weakening the attribution of treatment-control differences to the intervention. In many cases, results were under-analysed statistically. Follow-up investigations would add to the cost of evaluations although they would not usually involve biological markers or repeated surveys, the most expensive elements. The additional cost needs to be placed beside the cost of not being able to make proper sense of what happened: the Tehran sanitation study is an example (Kolahi *et al.* 2009).

### More responsive interventions

The endpoint of more realistic operational evaluations and more comprehensive SRs must be to support the development of interventions better able to contribute to people’s health and wellbeing. Implementers and stakeholders should be able to draw from these sources and experience in related contexts such as local management of irrigation systems (Meinzen-Dick and van der Hoek 2001; van Koppen *et al.* 2009) guidance on how the agency of beneficiaries and communities can be enlisted, from the design stage, and how their pursuit of multiple benefits can be accommodated. The institutional changes needed to achieve this should be illustrated, clarifying the choices available to governments and funders.

## Supplementary Data

Supplementary data are available at *Health Policy and Planning* online.

Supplementary Data

## Footnotes

^1^ Waddington and Snilstveit (2009) and Waddington *et al.* (2009) examine heterogeneity of the impact on diarrhoea in relation to several contextual variables such as season, baseline infrastructure status and compliance. The qualitative assessment of these factors is somewhat more extensive in the latter.

^2^ Kremer *et al.* (2009, p. 18); Waddington *et al.* (2009, Fig. 11).
