# Supplementary material for: The cost of a knowledge silo: a systematic re-review of water, sanitation and hygiene interventions
Source: Health Policy Plan. 2014 May 29;30(5):660–74. doi: 10.1093/heapol/czu039 (PMC4421832; doi:10.1093/heapol/czu039)
Supplement: Supplementary Data [file supp_30_5_660__index.html]

The cost of a knowledge silo: a systematic re-review of water, sanitation and hygiene interventions — The cost of a knowledge silo: a systematic re-review of water, sanitation and hygiene interventions — Supplementary Data 

# The cost of a knowledge silo: a systematic re-review of water, sanitation and hygiene interventions

## Supplementary Data

files

**Files in this Data Supplement:**

- Supplementary Data - doc file
- Supplementary Data - docx file
- Supplementary Data - doc file
- Supplementary Data - tiff file
- Supplementary Data - doc file
- Supplementary Data - doc file
- Supplementary Data - doc file
- Supplementary Data - doc file
- Supplementary Data - doc file
- Supplementary Data - doc file
- Supplementary Data - doc file
