# Supplementary material for: The cost of a knowledge silo: a systematic re-review of water, sanitation and hygiene interventions
Source: Health Policy Plan. 2014 May 29;30(5):660–74. doi: 10.1093/heapol/czu039 (PMC4421832; doi:10.1093/heapol/czu039)
Supplement: Supplementary Data [file supp_czu039_HPP444Table_3c_Knowledge_silo.doc]

| Context | Mechanism | Outcome | Implication for the diarrhoea outcome or its estimation in the study and the Waddington review |
| --- | --- | --- | --- |
| Agencies make operational decisions on where to site interventions and where to work first in situations of limited transparency and accountability. | These decisions may be affected by political influence, corruption and ease of access. Wealthier and healthier groups generally have greater influence and ability to offer bribes and live in more accessible, salubrious areas. | Interventions exacerbate existing inequality in services and health status. | Intervention’s effect is overestimated when comparing treatment and untreated areas without correction for baseline differences ; anti-poor distribution of benefits . |

Table 3c. Impact pathway related to unintended negative consequences of intervention
