# Supplementary material for: The cost of a knowledge silo: a systematic re-review of water, sanitation and hygiene interventions
Source: Health Policy Plan. 2014 May 29;30(5):660–74. doi: 10.1093/heapol/czu039 (PMC4421832; doi:10.1093/heapol/czu039)
Supplement: Supplementary Data [file supp_czu039_HPPOct444Supplement_Appendix__1_Knowledge_silo.docx]

**APPENDIX 1: STUDIES INCLUDED IN THE RE-REVIEW**

**Ahmed et al (1993)**

Ahmed, N.U., Zeitlin, M.F., Beiser, A.S., Super, C.M. and Gershoff, S.N., 1993. A

longitudinal study of the impact of behavioural change intervention on

cleanliness, diarrhoeal morbidity and growth of children in rural Bangladesh.

*Social science and medicine,* 37 (2), 159-171.

**Alam et al (1989)**

Alam, N., Wojtyniak, B., Henry, F.J. and Rahaman, M.M., 1989. Mothers'

personal and domestic hygiene and diarrhoea incidence in young children in

rural Bangladesh. *International journal of epidemiology,* 18 (1), 242-247.

**Aziz et al (1990)**

Aziz, K.M., Hoque, B.A., Hasan, K.Z., Patwary, M.Y., Huttly, S.R., Rahaman,

M.M. and Feachem, R.G., 1990. Reduction in diarrhoeal diseases in children in

rural Bangladesh by environmental and behavioural modifications. *Transactions*

*of the Royal Society of Tropical Medicine and Hygiene,* 84 (3), 433-438.

Aziz, K.M.A., Hoque, B.A., Huttly, S.R.A., Minnatullah, K.M., Hasan, Z., Patwary,

M.K., Rahaman, M.M. and Cairncross, S., 1990. *Water supply, sanitation and*

*hygiene education: report of a health impact study in Mirzapur, Bangladesh.*

Water and Sanitation report series, Washington, D.C.: UNDP-World Bank Water

and Sanitation Program.

Hasan, K.Z., Briend, A., Aziz, K.M., Hoque, B.A., Patwary, M.Y. and Huttly, S.R.,

1989. Lack of impact of a water and sanitation intervention on the nutritional

status of children in rural Bangladesh. *European journal of clinical nutrition,* 43

(12), 837-843.

Hoque, B.A., Juncker, T., Sack, R.B., Ali, M. and Aziz, K.M.A., 1996.

Sustainability of a water, sanitation and hygiene education project in rural

Bangladesh: a 5-year follow-up. *Bulletin of the World Health Organization*, 74

(4), 431-437.

**Bateman et al (1995)**

Bateman, O.M., Jahan, R.A., Brahman, S. and Zeitlyn, S. and Laston, S.L.,

1995. *Prevention of diarrhea through improving hygiene behaviors: the*

*Sanitation and Family Education (SAFE) Pilot Project experience.* Special

publication ICDDR,B, No. 42, Dhaka: International Centre for Diarrhoeal Disease

Research.

**Galiani et al (2007)**

Galiani, S., Gonzalez-Rozada, M. and Schargrodsky, E., 2007. *Water expansion*

*in shantytowns: Health and savings.* Inter-American Development Bank

Research Network working paper, No. PR-527, Washington D.C: Inter-American

Development Bank.

**Garrett et al (2008)**

Garrett, V., Ogutu, P., Mabonga, P., Ombeki, S., Mwaki, A., Aluoch, G., Phelan,

M. and Quick, R.E., 2008. Diarrhoea prevention in a high-risk rural Kenyan

population through point-of-use chlorination, safe water storage, sanitation, and

rainwater harvesting. *Epidemiology and infection,* 136 (11), 1463-1471.

**Gasana et al (2002)**

Gasana, J., Morin, J., Ndikuyeze, A. and Kamoso, P., 2002. Impact of water

supply and sanitation on diarrheal morbidity among young children in the

socioeconomic and cultural context of Rwanda (Africa). *Environmental research,*

90 (2), 76-88.

**Haggerty et al (1994)**

Haggerty, P.A., Manunebo, M.N., Ashworth, A., Muladi, K. and Kirkwood, B.R.,

1994a. Methodological approaches in a baseline study of diarrhoeal morbidity in

weaning-age children in rural Zaire. *International journal of epidemiology,* 23

(5), 1040-1049.

Haggerty, P.A., Muladi, K., Kirkwood, B.R., Ashworth, A. and Manunebo, M.,

1994b. Community based hygiene education to reduce diarrhoeal disease in

rural Zaire: impact of the intervention on diarrhoeal morbidity. *International*

*journal of epidemiology,* 23 (5), 1050-1059.

**Han and Hlaing (1989)**

Han, A.M. and Hlaing, T., 1989. Prevention of diarrhoea and dysentery by hand

washing. *Transactions of the Royal Society of Tropical Medicine and Hygiene,* 83,

128-131.

**Huttly et al (1990)**

Huttly, S.R., Blum, D., Kirkwood, B.R., Emeh, R.N., Okeke, N., Ajala, M., Smith,

G.S., Carson, D.C., Dosunmu-Ogunbi, O. and Feachem, R.G., 1990. The Imo

state (Nigeria) Drinking Water Supply and Sanitation Project, 2. Impact on

dracunculiasis, diarrhoea and nutritional status. *Transactions of the Royal*

*Society of Tropical Medicine and Hygiene,* 84 (2), 316-321.

Blum, D., Emeh, R.N., Huttly, S.R., Dosunmu-Ogunbi, O., Okeke, N., Ajala, M.,

Okoro, J.I., Akujobi, C., Kirkwood, B.R. and Feachem, R.G., 1990. The Imo state

(Nigeria) Drinking Water Supply and Sanitation Project, 1. Description of the

51 project, evaluation methods, and impact on intervening variables. *Transactions*

*of the Royal Society of Tropical Medicine and Hygiene,* 84 (2), 309-315.

**Jensen et al (2003)**

Jensen, P.K., Ensink, J.H., Jayasinghe, G., van der Hoek, W., Cairncross, S. and

Dalsgaard, A., 2003. Effect of chlorination of drinking-water on water quality and

childhood diarrhoea in a village in Pakistan. *Journal of health, population and*

*nutrition,* 21 (1), 26-31.

**Khan (1982)**

Khan, M.U., 1982. Interruption of shigellosis by handwashing. *Transactions of*

*the Royal Society of Tropical Medicine and Hygiene,* 76 (2), 164-168.

**Kolahi et al (2009)**

Kolahi, A.A., Rastegarpour, A. and Sohrabi, M.R., 2008. The impact of an urban

sewerage system on childhood diarrhoea in Tehran, Iran: a concurrent control

field trial. *Transactions of the Royal Society of Tropical Medicine and Hygiene*, 103, 500-505.

**Kremer et al (2009)**

Kremer, M. et al., 2009, Spring Cleaning: Rural Water Impacts, Valuation, and

Institutions. Available from:

<http://www.economics.harvard.edu/faculty/Kremer/files/SIP_2009.03.08-EMCLEAN.>

[pdf](http://www.economics.harvard.edu/faculty/Kremer/files/SIP_2009.03.08-EMCLEAN.) (accessed 7 July 2009).

**Lee et al (1991)**

Lee, W., Stoeckel, J., Jintaganont, P., Romanarak, T. and Kullavanijaya, S.,

1991. The impact of a community based health education program on the

incidence of diarrheal disease in southern Thailand. *Southeast Asian Journal of*

*tropical medicine and public health,* 22 (4), 548-556.

**Luby et al (2006)**

Luby, S.P., Agboatwalla, M., Painter, J., Altaf, A., Billhimer, W., Keswick, B. and

Hoekstra, R.M., 2006. Combining drinking water treatment and hand washing

for diarrhoea prevention, a cluster randomised controlled trial. *Tropical medicine*

*& international health,* 11 (4), 479-489.

**Luby et al (2005)**

Luby, S.P., Agboatwalla, M., Feikin, D.R., Painter, J., Billhimer, W., Altaf, A. and

Hoekstra, R.M., 2005. Effect of handwashing on child health: a randomised

controlled trial. *Lancet,* 366 (9481), 225-233.

Luby, S.P., Agboatwalla, M., Painter, J., Altaf, A., Billhimer, W.L. and Hoekstra,

R.M., 2004. Effect of intensive handwashing promotion on childhood diarrhea in

high-risk communities in Pakistan: a randomized controlled trial. *Journal of the*

*American medical association,* 291 (21), 2547-2554.

**Luby et al (2004)**

Luby, S.P., Agboatwalla, M., Hoekstra, R.M., Rahbar, M.H., Billhimer, W. and

Keswick, B.H., 2004. Delayed effectiveness of home-based interventions in

reducing childhood diarrhea, Karachi, Pakistan. *The American journal of tropical*

*medicine and hygiene,* 71 (4), 420-427.

**Moraes et al (2003)**

Moraes, L.R., Cancio, J.A., Cairncross, S. and Huttly, S., 2003. Impact of

drainage and sewerage on diarrhoea in poor urban areas in Salvador, Brazil.

*Transactions of the Royal Society of Tropical Medicine and Hygiene,* 97 (2), 153-

158.

**Pattanayak et al (2007)**

Pattanayak, S.K., Dickinson, K., Yang, J.C., Praharaj, P. and Poulous, C., 2007a.

*Promoting latrine use: Midline findings from a randomized evaluation of a*

*community mobilization campaign in Bhadrak, Orissa.* Working paper, No.

07_02, North Carolina: Research Triangle Institute.

Pattanayak, S.K., Dickinson, K., Yang, J.C., Patil, S.R., and Poulous, C., 2007b.

*Nature’s call Can social mobilization promote toilet use and improve welfare?*

*Results from a field experiment in Oeiss.* Draft paper, North Carolina: Research

Triangle Institute.

Pattanayak, S.K., Blitstein, J.L., Yang, J.C., Patil, S., Jones, K.M., Poulous, C.

and Dickinson, K., 2006. *Evaluating information and communication strategies*

*to promote latrine use and improve childe health: Design and baseline findings*

*from a community randomized trial in Bhadrak, Orissa.* Draft, North Carolina:

Research Triangle Institute.

**Pinfold and Horan (1996)**

Pinfold, J.V. and Horan, N.J., 1996. Measuring the effect of a hygiene behaviour

intervention by indicators of behaviour and diarrhoeal disease. *Transactions of*

*the Royal Society of Tropical Medicine and Hygiene,* 90 (4), 366-371.

**Shahid et al (1996)**

Shahid, N.S., Greenough, W.B.3., Samadi, A.R., Huq, M.I. and Rahman, N.,

1996. Hand washing with soap reduces diarrhoea and spread of bacterial

pathogens in a Bangladesh village. *Journal of diarrhoeal disease research,* 14

(2), 85-89.

**Sircar et al (1987)**

Sircar, B.K., Sengupta, P.G., Mondal, S.K., Gupta, D.N., Saha, N.C., Ghosh, S.,

Deb, B.C., Pal, S.C., 1987. Effect of hand washing on the incidence of diarrhoea

in a Calcutta slum. *Journal of Diarrhoeal Disease Research*, 5 (2), 112-114.

**Stanton et al (1988)**

Stanton, B.F., Clemens, J.D. and Khair, T., 1988. Educational intervention for

altering water-sanitation behaviour to reduce childhood diarrhea in urban

Bangladesh: impact on nutritional status. *American journal of clinical nutrition,*

48 (5), 1166-1172.

Stanton, B.F. and Clemens, J.D., 1987. An education intervention for altering

water-sanitation behaviours to reduce childhood diarrhea in urban Bangladesh

II. A randomized trial to assess the impact of the intervention on hygienic

behaviours and rates of diarrhea. *American journal of epidemiology,* 125 (2),

292-301.

Clemens, J.D. and Stanton, B.F., 1987. An educational intervention for altering

water-sanitation behaviors to reduce childhood diarrhea in urban Bangladesh I.

Application of the case-control method for development of an intervention.

*American journal of epidemiology,* 125 (2), 284-292.

**Tonglet et al (1992)**

Tonglet, R., Isu, K., Mpese, M., Dramaix, M. and Hennart, P., 1992. Can

improvements in water supply reduce childhood diarrhoea? *Health policy and*

*planning,* 7 (3), 260-268.

**Torun (1982)**

Torun, B., 1982. Environmental and educational interventions against diarrhoea

in Guatemala. *In*: L.C. Chen and N.S. Scrimshaw, eds. *Diarrhea and*

*malnutrition: interactions, mechanisms and interventions.* New York: Plenum

Press.

**Wilson et al (1991)**

Wilson, J.M., Chandler, G.N., Muslihatun and Jamiluddin, 1991. Hand-washing

reduces diarrhoea episodes: a study in Lombok, Indonesia. *Transactions of the*

*Royal Society of Tropical Medicine and Hygiene,* 85 (6), 819-821.

Wilson, J.M. and Chandler, G.N., 1993. Sustained improvements in hygiene

behaviour amongst village women in Lombok, Indonesia. *Transactions of the*

*Royal Society of Tropical Medicine and Hygiene,* 87 (6), 615-616.
