# Supplementary material for: The cost of a knowledge silo: a systematic re-review of water, sanitation and hygiene interventions
Source: Health Policy Plan. 2014 May 29;30(5):660–74. doi: 10.1093/heapol/czu039 (PMC4421832; doi:10.1093/heapol/czu039)
Supplement: Supplementary Data [file supp_czu039_HPPOct444Supplement_Appendix__2_Knowledge_silo.doc]

**Appendix 2 – Re-Review Form**

**Re-Review of Waddington et al. (2009) “Effectiveness and sustainability of water, sanitation, and hygiene interventions in combating diarrhoea”**

NB. Please feel free to cut and paste passages from the paper to substantiate your answers

Reviewer name:

1. Author/year/title

2. Remarks on paper in Waddington et al.

3. Intervention as categorized in Waddington et al

4. From the paper: what else was done, besides the intervention listed in (3), that might have had an effect on the benefits experienced? Please specify whether this action(s) was contemporary with the intervention or in the recent past, undertaken by the same organization or a different one (including people/communities themselves).^1^

5. **Judgment**: Is the intervention substantially more complex than considered by Waddington et al.? Please rank by likelihood, using the guide below:

No evidence that it is substantially more complex ____;

Possible that it is ____; More than possible that it is ____; Likely that it is ____

Possible: Several factors need to be present, each not very probable;

More than possible: Fewer factors need to be present, each quite probable;

Likely: One or only a few factors need to be present, each probable.

6. Are there benefits or disbenefits associated with the intervention(s), other than diarrhoea reduction, that emerge from the paper? Please describe, including who experiences them.

7. **Judgment**: Is the range of impacts of the intervention(s) substantially understated if only the effect on diarrhoea is considered? Please rank by likelihood, using the guide above:

No evidence that it is substantially understated ____;

Possible that it is ____; More than possible that it is ____; Likely that it is ____

8. Are these benefits/disbenefits: assessed and discussed by the authors ____ ; mentioned or even measured e.g. in the baseline characterization of sites/subjects, but not discussed further ______ ; suggested by the description of setting and context, or from the discussion section but not considered by the authors ____?

9. Are these benefits/disbenefits visible to those who supposedly experience them?

10. Are these benefits/disbenefits uncertain from the perspective of those who experience them?

11. **Judgment**: Are actions by individuals, households or communities[[1]](#footnote-2) substantially influencing the benefits and disbenefits experienced?[[2]](#footnote-3) Please specify whose actions:

and rank by likelihood, using the guide above:

No evidence that actions are influencing benefits/disbenefits ____;

Possible that they are ____; More than possible that they are ____; Likely that they are ____

12. **Judgment**: Would these other benefits/disbenefits and actions substantially affect, positively or negatively, the level, distribution or sustainability of the diarrhoea morbidity outcome? How?

and rank by likelihood, using the guide above:

No evidence that the diarrhoea outcome would be affected ____;

Possible that it would be ____; More than possible that it would be ____; Likely that it would be ____

1. Question 11 asks about people’s actions that affect the benefits/disbenefits experienced from interventions e.g. social pressure influencing open defecation after the village was initiated into CLTS or communities organizing to maintain pumps an agency installed. In contrast, question 4 asks about interventions that people initiate alongside, after or before an organization-led intervention e.g. a village not initiated undertaking CLTS on its own or households outside a water supply project installing their own pumps. [↑](#footnote-ref-2)
2. Actions that go beyond those assumed in the intervention’s program theory e.g. people washing their hands in a hygiene program promoting soap use. [↑](#footnote-ref-3)
