# Supplementary figures and images for: The cost of a knowledge silo: a systematic re-review of water, sanitation and hygiene interventions
Source: Health Policy Plan. 2014 May 29;30(5):660–74. doi: 10.1093/heapol/czu039 (PMC4421832; doi:10.1093/heapol/czu039)

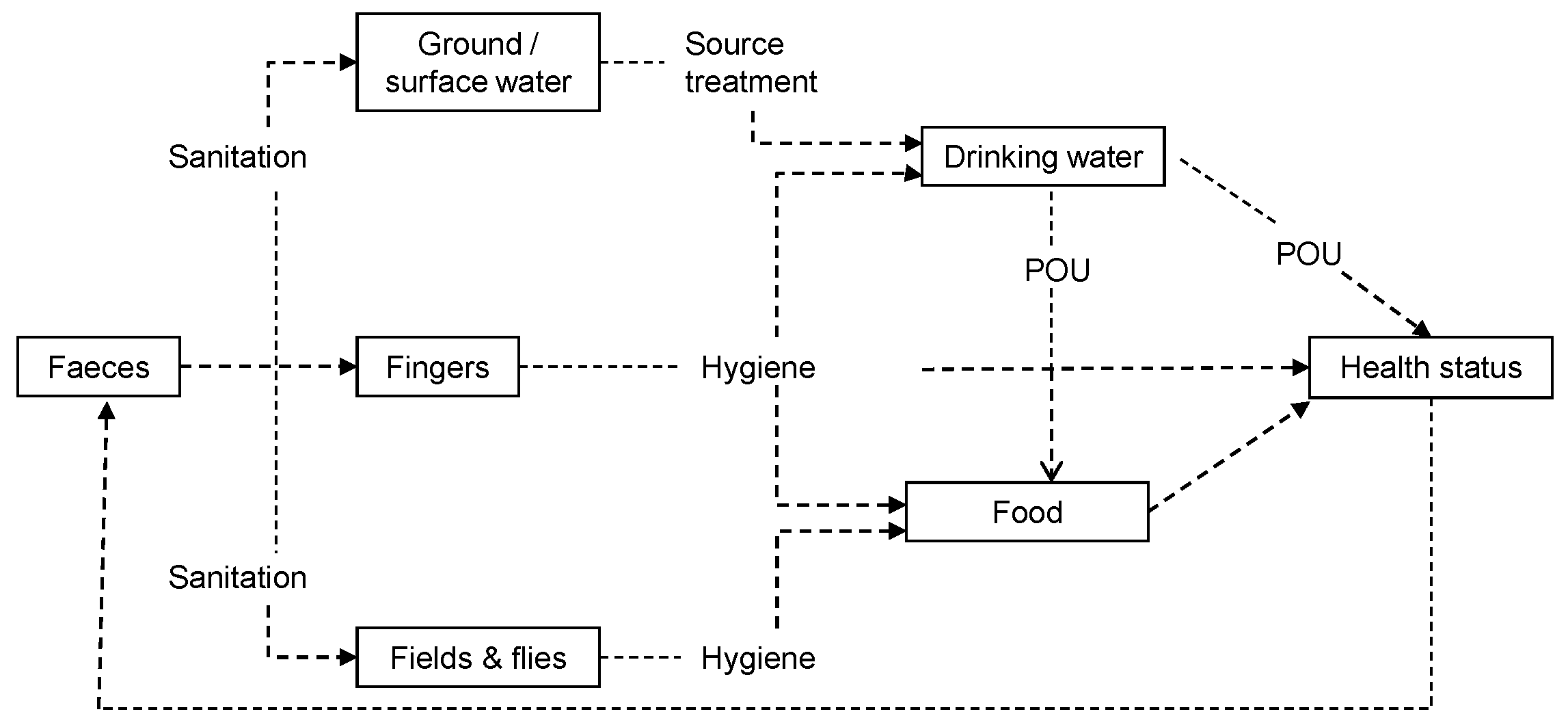

Supplement: Supplementary Data [file supp_czu039_Knowledge_silo_FIGURE_1.tif]
