# Supplementary material for: The cost of a knowledge silo: a systematic re-review of water, sanitation and hygiene interventions
Source: Health Policy Plan. 2014 May 29;30(5):660–74. doi: 10.1093/heapol/czu039 (PMC4421832; doi:10.1093/heapol/czu039)
Supplement: Supplementary Data [file supp_czu039_Table_1_Knowledge_silo.doc]

Table 1. Reassessment of studies from the Waddington review

|  | **Is the intervention substantially more complex than considered by the Waddington review?** | **Are impacts substantially understated if only diarrhoea outcome is considered?** | **Are actions by individuals, households or communities substantially influencing the benefits and harms experienced?** | **Would these other impacts and actions substantially affect the level, distribution or sustainability of the diarrhoea outcome?** |
| --- | --- | --- | --- | --- |
| **Likely** | 5 (18.5) | 6 (22.2) | 5 (18.5) | 5 (18.5) |
| **More than possible** | 1 (3.7) | 4 (14.8) | 8 (29.6) | 7 (25.9) |
| **Possible** | 4 (14.8) | 1 (3.7) | 3 (11.1) | 4 (14.8) |
| **No evidence** | 17 (63.0) | 16 (59.3) | 11 (40.7) | 11 (40.7) |

N = 27. Data are number (%)
