# Supplementary material for: The cost of a knowledge silo: a systematic re-review of water, sanitation and hygiene interventions
Source: Health Policy Plan. 2014 May 29;30(5):660–74. doi: 10.1093/heapol/czu039 (PMC4421832; doi:10.1093/heapol/czu039)
Supplement: Supplementary Data [file supp_czu039_Table_2_Knowledge_silo.doc]

Table 2. Reassessment of hygiene interventions in the Waddington review: prevalence of affirmative judgments at the “more than possible” or “likely” level

|  | **Is the intervention substantially more complex than considered by the Waddington review?** | **Are impacts substantially understated if only diarrhoea outcome is considered?** | **Are actions by individuals, households or communities substantially influencing the benefits and harms experienced?** | **Would these other impacts and actions substantially affect the level, distribution or sustainability of the diarrhoea outcome?** |
| --- | --- | --- | --- | --- |
| **Hygiene only (n=13)** | 2 (15.4) | 2 (15.4) | 4 (30.8) | 4 (30.8) |
| **Other interventions (n=14)** | 4 (28.6) | 8 (57.1) | 9 (64.3) | 8 (57.1) |

Data are number (%)
