# Supplementary material for: The cost of a knowledge silo: a systematic re-review of water, sanitation and hygiene interventions
Source: Health Policy Plan. 2014 May 29;30(5):660–74. doi: 10.1093/heapol/czu039 (PMC4421832; doi:10.1093/heapol/czu039)
Supplement: Supplementary Data [file supp_czu039_Table_3a_Knowledge_silo.doc]

Table 3a Impact pathways related to intervention complexity

| Context | Mechanism | Outcome | Implication for the diarrhoea outcome or its estimation in the study and the Waddington review |
| --- | --- | --- | --- |
| Agencies make operational decisions on what to include in their intervention and where to intervene. | (a) Staff modify intervention in response to local circumstances | (a) The intervention implemented differs substantially from the label, involving additional elements that affect its impact | Effect of the (current) intervention is overestimated |
|  | (b) Interventions cluster in certain areas to draw on the information from earlier efforts or in response to policy or administrative directive | (b) What people experience includes the contribution of both the current and earlier interventions |  |
