# Supplementary material for: The cost of a knowledge silo: a systematic re-review of water, sanitation and hygiene interventions
Source: Health Policy Plan. 2014 May 29;30(5):660–74. doi: 10.1093/heapol/czu039 (PMC4421832; doi:10.1093/heapol/czu039)
Supplement: Supplementary Data [file supp_czu039_Table_3b_Knowledge_silo.doc]

| Context | Mechanism | Outcome | Implication for the diarrhoea outcome or its estimation in the study and the Waddington review |
| --- | --- | --- | --- |
| Several sources of ill-being, including non-diahorreal infections, are linked to poor access to water and to insanitary environment. | WASH interventions alleviate determinants of these different sources of ill-being. | Multiple benefits (health, time and expense saved by more accessible services), in addition to diarrhoea reduction, may be realized as a direct consequence of the intervention . | Possibly no effect (additional benefits are valued in their own right); may increase beneficiaries’ commitment to supporting and maintaining the intervention, enhancing sustainability. |

Table 3b. Impact pathway related to the direct multiple benefits of interventions
