# Supplementary material for: The cost of a knowledge silo: a systematic re-review of water, sanitation and hygiene interventions
Source: Health Policy Plan. 2014 May 29;30(5):660–74. doi: 10.1093/heapol/czu039 (PMC4421832; doi:10.1093/heapol/czu039)
Supplement: Supplementary Data [file supp_czu039_Table_3d_Knowledge_silo.doc]

Table 3d. Impact pathway linking domestic water supply to food production or purchase

| Water supply interventions enable beneficiaries not only to avoid water-related diseases but also to access a resource, increasingly in demand, that can be used for a range of purposes. | People often use water for production (esp. of food in rural/peri-urban areas); or to reduce private expenditure for water. The poor use the additional income in large proportion to purchase food. They also save time procuring water. | Increased food, water and time are valued in their own right. Improved child nutrition may also contribute to reduced diarrhoea. | Attribution of diarrhoea reduction solely to direct effect of water supply may be mistaken; people’s commitment to support and maintain the system is increased, enhancing sustainability (Aziz et al., 1990a,b; Hoque et al., 1996); markedly pro-poor distribution of benefits is unrecognized . |
| --- | --- | --- | --- |
