# Supplementary material for: The cost of a knowledge silo: a systematic re-review of water, sanitation and hygiene interventions
Source: Health Policy Plan. 2014 May 29;30(5):660–74. doi: 10.1093/heapol/czu039 (PMC4421832; doi:10.1093/heapol/czu039)
Supplement: Supplementary Data [file supp_czu039_Table_3e_Knowledge_silo.doc]

Table 3e. Impact pathway related to the diffusion of innovations

| Interventions are implemented in communities whose members are linked in social networks; they are also linked, generally less intensively, with people in neighbouring communities. | Information that people gain from interventions and their experience with new practices moves through these networks. | Information from interventions or direct access to infrastructure benefits people in control as well as treatment groups. | Estimates of diarrhoea morbidity reduction based on the difference between treatment and control groups are biased downwards . |
| --- | --- | --- | --- |
