# Supplementary material for: The cost of a knowledge silo: a systematic re-review of water, sanitation and hygiene interventions
Source: Health Policy Plan. 2014 May 29;30(5):660–74. doi: 10.1093/heapol/czu039 (PMC4421832; doi:10.1093/heapol/czu039)
Supplement: Supplementary Data [file supp_czu039_Table_3f_Knowledge_silo.doc]

Table 3f. Impact pathway related to local institutions

| Context | Mechanism | Outcome | Implication for the diarrhoea outcome or its estimation in the study and the Waddington review |
| --- | --- | --- | --- |
| The communities in which interventions are implemented have an adaptive capacity for self-governance. | Local institutions, formal and informal, influence the spread, adaptation and retention of interventions. | Distribution of benefits and their sustainability are generally positively affected. | The effect of institutions on distribution and sustainability of the diarrhoea morbidity reduction is largely unrecognized by the systematic review . |
